# Femoral Head Avascular Necrosis Due to Brucella Infection: A Case Report

**DOI:** 10.7759/cureus.65740

**Published:** 2024-07-30

**Authors:** Binar B Abdulrahman, Botan N Sadq, Farman U Shareef, Jeza M Abdul Aziz, Mariwan K Rasheed

**Affiliations:** 1 Biomedical Sciences, Komar University of Science and Technology, Sulaymaniyah, IRQ; 2 Medical Microbiology, Koya University, Sulaymaniyah, IRQ; 3 Physiotherapy, Tishk International University Sulaimani, Sulaymaniyah, IRQ; 4 Medical Laboratory Science, Charmo University, Sulaymaniyah, IRQ; 5 Research Center, Baxshin Hospital, Sulaymaniyah, IRQ; 6 Medical Laboratory Science, University of Human Development, Sulaymaniyah, IRQ

**Keywords:** fhavn, cas report, arthritis, avascular necrosis femoral head, brucellosis

## Abstract

Brucellosis remains a widespread disease in endemic regions worldwide and is not adequately controlled. It is a common zoonotic disease worldwide, a systemic infection, and a major health problem in endemic countries. Femoral head avascular necrosis (FHAVN) as a consequence of brucellosis is exceedingly unusual and has seldom been recorded. The case reports a 21-year-old female patient was hospitalized due to severe pain in both lower limbs, particularly in the anterior portion of the hip joint, accompanied by a low-grade fever persisting for six months. Movement of the right hip was painful, and the patient limped at the beginning of walking after a few steps. Rheumatoid factor and antinuclear antibody test results were negative. The right hip joint was aspirated, and a small quantity of fluid was sent for Gram staining and culture. Synovial joint fluid culture confirmed Brucella abortus infection after four weeks. The source of infection in the present case was the consumption of raw milk. Based on laboratory tests and radiographic images, FHAVN was diagnosed. Owing to misdiagnosis, she had not received standard treatment for brucellosis in the previous months. The patient was diagnosed early, and she was in the third stage. After the patient received medical treatment, the left and right hip joints partly recovered. The right hip joint required replacement; however, the patient refused. Attending physicians should consider brucellosis as an alternative to arthritis for hip joint pain in Brucella-endemic locations. Medication-based therapy may be effective for early avascular necrosis, emphasizing the need for early diagnosis and treatment.

## Introduction

Brucellosis is a bacterial infection caused by Brucella species. It is a common zoonotic disease worldwide, a systemic infection, and a major health problem in endemic countries. It is usually transmitted from animals to humans and can infect any organ or system in the body [1]. Brucellosis primarily affects the musculoskeletal system [2]. Femoral head avascular necrosis (FHAVN) is a condition characterized by the death of bone cells in the femoral head due to inadequate blood supply, leading to the collapse of the joint and it causes excruciating agony; makes walking difficult; and invariably necessitates total hip replacement (THR), while the specific cause of FHAVN is unknown [3,4].

Specifically, 93% of avascular necrosis of the femoral head cases are of unknown causes, but alcoholism, steroid use, and traumatic events are mentioned among the etiological factors. Brucella, which affects phagocytic cells, is involved in the pathogenesis of osteoarticular system complications and avascular necrosis that develop due to interference with the vascular structure of the bone [5]. Bacterial osteonecrosis of the femoral head is an uncommon condition that occurs when bacteria invade the area and block the blood vessels [6]. The present study highlights brucellosis as a potential cause of bilateral FHAVN in endemic regions.

## Case presentation

A 21-year-old female patient was hospitalized due to severe pain in both lower limbs, particularly in the anterior portion of the hip joint, accompanied by a low-grade fever persisting for six months. The situation has worsened over the past month, with symptoms progressively increasing. She had lost weight (10 kg) and had a body mass index (BMI) of 18.7. She was taking intermittent painkillers, such as non-steroidal anti-inflammatory drugs (NSAIDs) (7.5 mg) once daily and antipyretics (paracetamol 1 g every six hours, as necessary) for six months but did not show any improvement. The patient did not consume corticosteroids, alcohol, or smoke. She had a healthy appetite, always consumed an adequate diet, and consumed only small amounts of unpasteurised cow milk. Her family and medical histories were normal.

Movement of the right hip was painful, with a temperature of 37.8 °C, tachycardia at 110 bpm, respiration rate (18/mint), and blood pressure (119/80 mmHg). On physical examination, she had an antalgic gait, limping, thin, and ill-looking, with a weight loss of approximately 10 kg in six months, and limited movement in both hip joints, particularly with internal and external rotation. She refused to put weight on it. No palpable lymph nodes were observed. The spine examination was normal, apart from the painful straight leg raising test, hip range of motion (ROM) flexion at 100 degrees, extension at 30 degrees, external rotation at 25 degrees, and internal rotation at 30 degrees. She limped at the beginning of walking after a few steps. Spinal examination results were normal.

At the time of admission, laboratory tests showed the following: hemoglobin 11.5 g/dL; white blood cell count, 7 × 109/µL; and platelet count, 278 × 109/µL. The erythrocyte sedimentation rate (ESR) was 82 mm/hour (Ves matic20), and C-reactive protein (CRP) was 7.8 mg/L (Cobas C3111), Brucella IgG was 0.2 (negative), and Brucella IgM was 12 (positive)(ELISA, Biotek) (Table 1).

**Table 1:** Laboratory tests

Laboratory Tests	Pretreatment	Treatment (2 months)	Treatment (6 months)	Normal range
White blood cell count	7×10^9^/µL	7.2×10^9^/µL	5.4×10^9^/µL	3.5-10×10^9^/µL
Hemoglobin	11.5g/dL	11.7g/dl	12.2g/dl	11.5-16.5g/dL
Platelet count	287×10^9^/µL	300×10^9^/µL	303×10^9^/µL	100-400×10^9^/µL
Erythrocyte sedimentation rate (ESR)	82 mm/hour	55 mm/hour	11 mm/hour	17-50 years, female <12 mm/hour
C-reactive protein (CRP)	7.8 mg/L	5.7 mg/L	3.76 mg/L	<5 mg/L
Brucella IgG	0.2 IU/mL	0.2 IU/mL	0.2 IU/mL	< 9 IU/mL, negative; >11 IU/mL, positive
Brucella IgM	12 IU/mL	10 IU/mL	9.1 IU/mL	
				< 9 IU/mL, negative; >11 IU/mL, positive

Rheumatoid factor and antinuclear antibody test results were negative. The right hip joint was aspirated, and a small quantity of fluid was sent for Gram staining and culture. Synovial joint fluid culture confirmed Brucella abortus infection after four weeks.

**Figure 1:**
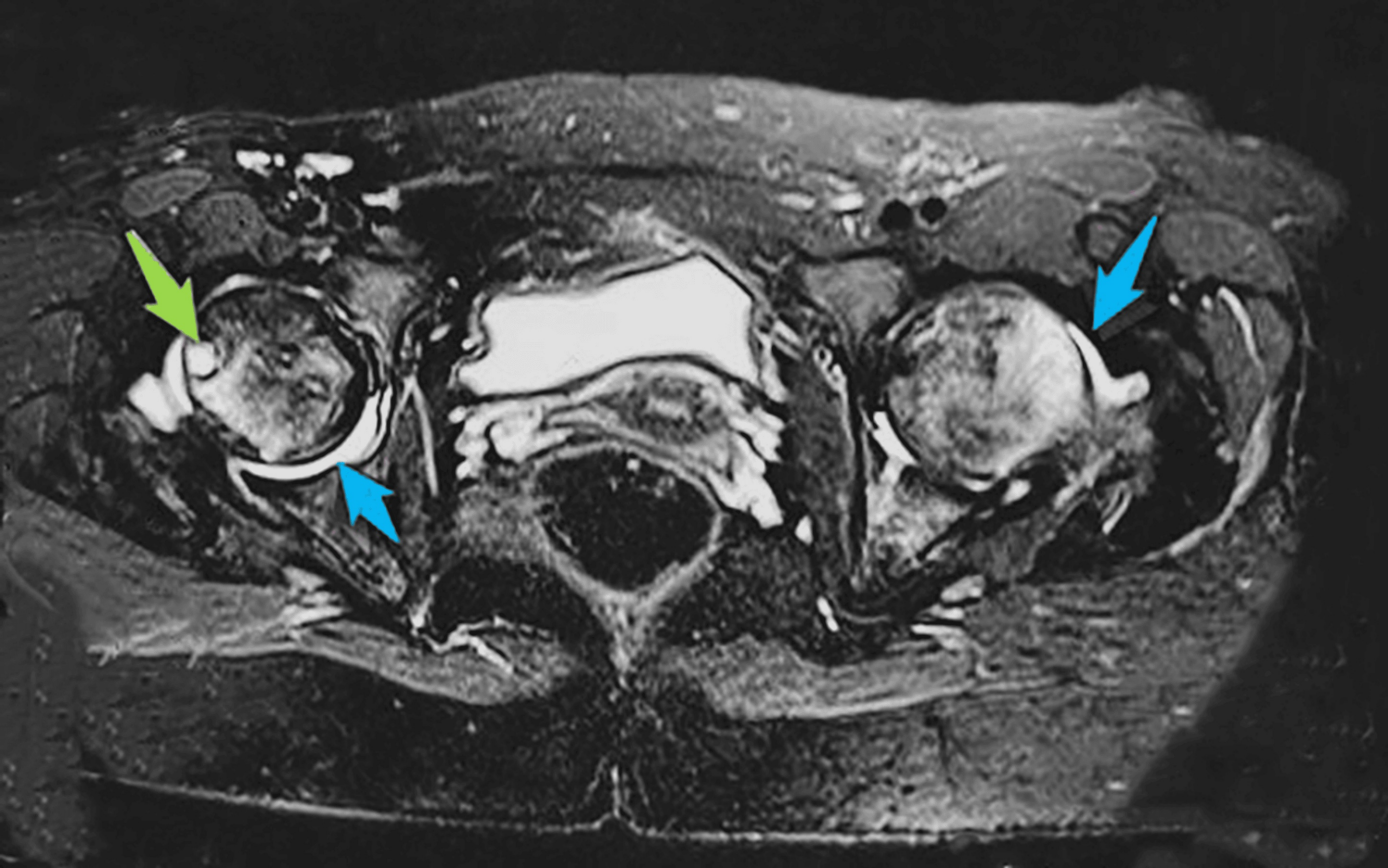
Bilateral hip MRI indicating joint effusion (blue arrow) and a subchondral cyst on the right side (green arrow)

The patient underwent imaging studies, including radiography of the pelvis with both hips and magnetic resonance imaging (MRI). Radiography revealed subchondral lesions in both joints. MRI revealed bone marrow edema in the femoral head and neck, mild joint effusion bilaterally, and a subchondral cyst on the right side (Figure 1).

**Figure 2:**
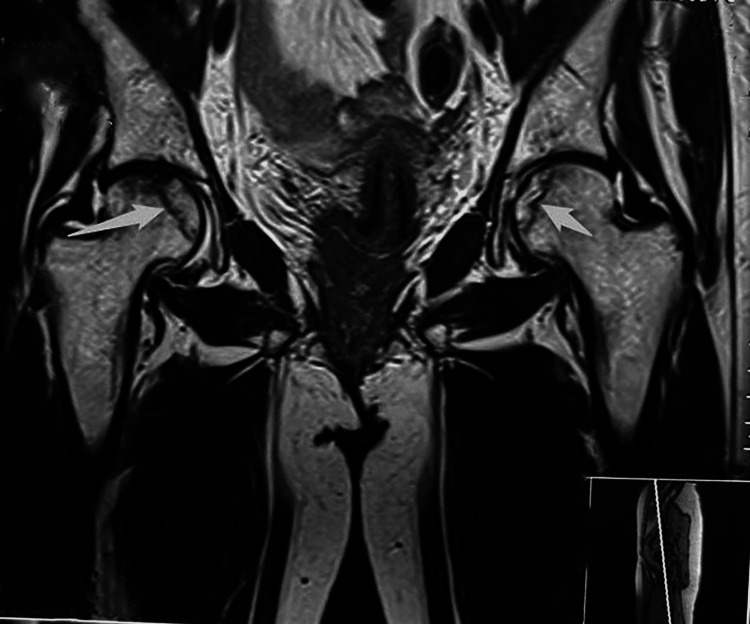
MRI of the pelvis showed bilateral femur head avascular necrosis (white arrow)

The right femoral head appeared diffusely hypo-enhanced compared to the left, raising concerns about impending avascular necrosis (stage III) (Figure 2).

Normal peri-articular musculature with preserved intervening fat plane

Owing to misdiagnosis, she had not received standard treatment for brucellosis in the previous months. After diagnosis, the patient received antibiotics, including doxycycline (100 mg orally, twice a day for 10 weeks), rifampin (600 mg, once a day for four weeks intramuscular, continued on rifampicin tab 300 mg for six weeks), and intramuscular streptomycin 1 g for six weeks [2,7,8]. In the six-month treatment, the patient’s ESR decreased to 11 mm/hour, as the fever quickly subsided. However, a follow-up MRI revealed moderate avascular necrosis of the right femoral head, and the patient declined hip replacement. The patient could walk normally and perform most activities of daily living, except for pain, while sitting on the ground. Therefore, replacement was postponed until the patient decided. Six months and one year of follow-up, MRI showed improvement in edema and fluid accumulation within the joint. Nevertheless, signs of AVN persisted.

## Discussion

Brucellosis remains a widespread disease in endemic regions worldwide and is not adequately controlled [2,3]. Specifically, 30-85% of brucellosis patients report osteoarticular symptoms, such as arthritis, spondylitis, sacroiliitis, osteomyelitis, tendinitis, and bursitis [9]. In recent decades, emerging diseases in humans have originated in animals and are directly related to diets derived from animals [10]. The source of infection in this case was the consumption of raw milk. Therefore, a patient's history is helpful for physicians in making a diagnosis [11].

The spread of Brucella through the blood to the hip joints and other large joints leads to the disruption of blood flow to a particular region of the femoral head, which gradually becomes necrotic. Ischemia, direct cell toxicity, and altered development of mesenchymal stem cells are some of the current pathogenic causes of bone cell death, and the incidence of Brucella arthritis among patients with osteoarticular brucellosis ranges from 18 to 60% [3,10].

As the patient had no history of corticosteroid use or alcohol misuse, the physician assumed that the femoral head necrosis was caused by brucellosis. Researchers recommend extending the incubation period of three to four weeks for bacterial cultures from synovial fluid to enhance diagnostic accuracy [7]. In the present case, synovial fluid culture revealed brucellosis after four weeks. Previously, conventional radiography was used to diagnose FHAVN. Recently, MRI has become the gold standard method for early diagnosis and determination of the best course of action [12,13]. Depending on age, the severity of pain/discomfort, location and extent of necrosis, comorbidities, and articular surface collapse of the disease determine the surgical therapy options. Core decompression is a successful treatment for the pre-collapse stages, whereas late stages 3 and 4 may require hip replacement surgery [4,12]. The current case was diagnosed late, and the stage was the third stage; however, after the patient was under medical treatment, the infection was controlled. However, the right hip joint required replacement and the patient refused.

## Conclusions

When a patient complains of discomfort in the hip joint, the attending physician should consider brucellosis as a possible alternative diagnosis to arthritis, particularly in areas where Brucella infection is endemic. Non-operative treatment with medication-based therapy may be effective in the early stages of avascular necrosis, emphasizing the need for early diagnosis and prompt intervention.
